# Topical vs. intravenous administration of tranexamic acid in knee arthroplasty and prevalence of deep venous thrombosis: a randomized clinical trial

**DOI:** 10.1590/1677-5449.007515

**Published:** 2016

**Authors:** Ari Zekcer, Ricardo Del Priori, Clauber Tieppo, Ricardo Soares da Silva, Nilson Roberto Severino

**Affiliations:** 1 Clínica Ortopédica Tatuapé, São Paulo, SP, Brazil.; 2 Clínica Santa Maria, São Paulo, SP, Brazil.; 3 Instituto de Ortopedia Campo Belo, São Paulo, SP, Brazil.; 4 Clínica Ortopédica Tatuapé, São Paulo, SP, Brazil.; 5 Faculdade de Ciências Médicas – FCM, Santa Casa de São Paulo, São Paulo, SP, Brazil.

**Keywords:** tranexamic acid, arthroplasty, knee replacement, fibrin modulating agents, antifibrinolytic agents, venous thrombosis, ácido tranexâmico, artroplastia, artroplastia do joelho, moduladores de fibrina, antifibrinolíticos, trombose venosa

## Abstract

**Background:**

Tranexamic acid (TXA) is widely used in orthopedic surgery to reduce perioperative bleeding. Since TXA inhibits fibrinolysis, there is concern that it may increase the risk of thromboembolic events.

**Objectives:**

To verify the prevalence of deep venous thrombosis (DVT) in patients receiving TXA during total knee arthroplasty and to compare topical with intravenous administration of the drug.

**Methods:**

All patients admitted for total knee arthroplasty due to primary arthrosis between June and November of 2014 were recruited consecutively. Thirty patients were randomized to a “topical group” (1.5 g TXA diluted in 50ml saline sprayed over the area operated, before tourniquet release), 30 to an “intravenous group” (20mg/kg TXA in 100 ml of saline, given at the same time as anesthesia), and 30 to a control group (100 ml of saline, given at the same time as anesthesia). All patients had duplex ultrasound scans of the legs on the 15th postoperative day.

**Results:**

Deep venous thrombosis events occurred in five of the 90 patients operated (one out of 30 in the topical group [3.3%], four out of 30 in the control group [13.3%], and zero in the intravenous group). All were confirmed by duplex ultrasound scans and all were asymptomatic. Prevalence rates of DVT were similar between groups (p = 0.112 for control vs. intravenous; p = 0.353 for control vs. topical; and p =1.000 for intravenous vs. topical, according to two-sided exact tests).

**Conclusions:**

Both topical and intravenous administration of TXA are safe with regard to occurrence of DVT, since the number of DVT cases in patients given TXA was not different to the number in those given placebo.

## Resumo

**Contexto:**

O ácido tranexâmico é amplamente utilizado em cirurgia ortopédica para reduzir a hemorragia perioperatória. Como o ácido tranexâmico inibe a fibrinólise, há uma preocupação de que ele possa aumentar o risco de eventos tromboembólicos.

**Objetivos:**

Verificar se o uso do ácido tranexâmico é seguro em relação à prevalência de trombose venosa profunda em pacientes submetidos a artroplastia total do joelho, e comparar as administrações tópica e intravenosa desse medicamento.

**Métodos:**

Todos os pacientes consecutivamente admitidos para artroplastia total do joelho devido a artrose primária entre junho e novembro de 2014 foram recrutados. Os pacientes foram randomizados em um “grupo tópico” (1,5 g de ácido tranexâmico diluído em 50 ml de solução salina cobrindo toda a área operada antes de liberar o torniquete), um “grupo intravenoso” (20 mg/kg de ácido tranexâmico em 100 ml de solução salina no momento da anestesia) e um “grupo controle” (100 ml de solução salina com a anestesia). No 15º dia de pós-operatório, todos os pacientes foram submetidos a ultrassonografia vascular com Doppler de membros inferiores, independentemente de sintomas.

**Resultados:**

Dos 90 pacientes operados, apenas cinco apresentaram trombose venosa profunda (um no grupo tópico e quatro no grupo controle).

**Conclusões:**

Tanto a administração tópica quanto a intravenosa de ácido tranexâmico são seguras em termos de ocorrência de trombose venosa profunda, pois o número de casos de trombose venosa profunda foi semelhante quando comparamos os pacientes que receberam ácido tranexâmico e os que receberam placebo. Novos estudos, com amostras maiores, são necessários para confirmar esse achado.

## INTRODUCTION

The nature of orthopedic and traumatic disorders and surgical treatment, especially in hip and knee surgeries, means that they involve a high risk of venous thromboembolism.^1^ Positioning of the limb operated during total hip surgery (THA) and total knee arthroplasty (TKA), edema at the site operated, and limited mobility in the immediate postoperative period can all contribute to venous stasis, with a consequent reduction in blood flow.^2^ Secondary thermal reaction to use of cement and other tissue injuries may also activate thrombogenic factors that lead to tropism in areas of vascular injury and stasis. Likewise, blood loss can reduce antithrombin III levels and inhibit the endogenous fibrinolytic system, allowing thrombus formation and growth at all levels.^3^^-^ 5 Surgical trauma in TKA induces fibrinolysis^6^^-^ 9 and coagulation activation,^10^^,^ 11 especially when a pneumatic tourniquet is used.^9^^,^ 12 ^-^ 14

Research into hemostatic and anti-thrombolytic drugs began after World War II. Use of tranexamic acid (TXA) for excessive bleeding after tonsillectomy was initiated in Japan in 1965.^6^^,^ 10 ^,^ 15 ^-^ 17 Tranexamic acid inhibits fibrinolysis by competing with the lysine molecule in coupling sites in fibrinogen,^18^^,^ 19 reducing bleeding during surgery, which varies between 800 ml and 1800 ml if an antifibrinolytic is not used.^6^^,^ 10 ^,^ 15 ^-^ 17 Tranexamic acid has been used successfully in cardiac surgery (mainly revascularizations),^18^^,^ 20 orthotopic organ transplantations,^21^ gynecological surgery (uterus),^22^ and orthopedic surgery (e.g. total knee replacement).^23^ A recent metanalysis evaluated TXA use in emergency surgeries (trauma and cardiac surgeries) and concluded that the drug reduces blood transfusion in the emergency setting too.^24^

However, despite successful use of TXA for reducing perioperative bleeding, including in lower limb surgery, there are still concerns over the increased risk of thromboembolic events. It has been shown that preoperative administration of TXA does not increase the incidence rates of deep vein thrombosis (DVT) or pulmonary embolism (PE) in primary total knee (TKR) or total hip replacement (THR).^11^ As shown in a recent study published by Schiff et al., peak incidence of DVT after TKR occurs between the fourth and the eighth postoperative day, and is greatest on the sixth day.^25^ In a recent metanalysis, Ker et al.^26^ analyzed the effect of topical TXA on bleeding and DVT incidence. In studies by Wong et al.^27^ and Sa-Ngasoonsong et al.^28^ that were reviewed in the metanalysis, duplex ultrasound scanning was used in all patients on the second and third postoperative days and on the fourth postoperative day, respectively, and no significant increase in DVT incidence was observed. It is important to observe, however, that the ultrasound examinations were performed during the period in which patients were still on anticoagulant therapy, and before the known peak incidence of DVT on the sixth day. In studies by Ishida et al.^29^ and Alshryda et al.^30^ (also reviewed by Ker et al.^26^), duplex ultrasound scanning was performed only for patients exhibiting symptoms.

The objective of this randomized, controlled, clinical trial was to verify whether use of TXA is safe with regard to the prevalence of DVT. The aim was therefore to evaluate DVT prevalence in patients undergoing TKR and given either intravenous or topical TXA, using duplex ultrasound scanning in all patients. The hypothesis was that the prevalence of DVT would be the same for topical and intravenous administrations of the drug.

## MATERIAL AND METHODS

### Trial design, interventions and setting

This is a randomized, single-blinded, controlled clinical trial, conducted between June and November 2014 at a regional referral center for orthopedics in Brazil. The study protocol (number 27270814.0.0000.0085) was approved by the hospital's institutional ethics committee, and the patients signed informed consent forms before surgery. The study protocol is registered at clinicaltrials.gov (protocol record NCT02323373) and on the Plataforma Brasil (CAAE: 27270814.0.0000.0085).

All consecutive patients recruited during the period were randomized to one of three groups. Patients in the “topical group” (*n* = 30) were administered a solution of 1.5 g of TXA (50 mg/ml, Transamin®, Zydus Nikkho) diluted in 50 ml of saline (at 0.9%), which was sprayed over the operated area for 5 minutes, before the tourniquet was released. Patients in the “intravenous group” (*n* = 30) were administered 20 mg/kg of TXA, diluted in 100 ml of saline at 0.9%, over a 10-minute period at the same time as anesthesia was administered. Patients in the control group (*n* = 30) were only administered 100 ml of saline solution, also at the same time as anesthesia, over a period of 10 minutes.

All patients were blinded to their treatment group. However, for technical reasons the surgical team and the anesthesiologists were aware of treatment allocation. The anesthesiologist was responsible for the randomization procedure, after sedation in the operating room, in order to guarantee that the patients were blinded to allocation. Randomization was conducted using previously prepared and sealed opaque envelopes, with an allocation ratio of 1:1:1.

### Inclusion of participants and general surgical care

The patients included in this study all had indications for unilateral TKA due to arthrosis (Albach grades III and IV) and were recruited at the study authors’ private clinics. All were treated using the same surgical technique: total resection of the posterior cruciate ligament, patellar arthroplasty, and closure of the femoral canal with a bone plug. Lateral release of the patella was not performed in any of the patients. All patients were fitted with the same type of prosthesis (Genesys 2®, Smith & Nephew, Memphis, USA) by the same surgery team (all experienced knee surgeons, with no residents among them) and with the same anesthesiology team, using standardized care.

Patients were excluded if they had previously undergone any orthopedic surgery to the legs or if they had secondary arthrosis. Other exclusion criteria were: a history of DVT or PE or identified risks for DVT or PE, coagulation or cardiovascular disorders, or vascular diseases. Patients were also excluded if they were currently using anticoagulation drugs. Preoperative hemoglobin was recorded.

General anesthesia (propofol, fentanyl and cisatracurium besylate for tracheal intubation) was used only when spinal puncture (double block, with 4 ml of bupivacaine 0.5%, epidural catheter) was not possible (for example, because of a previous spinal arthrodesis or other anatomical problems preventing punctures). Bolus intravenous hydration (8 ml/kg) was administered immediately before surgery and 4 ml/kg/hour of saline were administered during surgery. Tourniquets were routinely used on all patients and were released after application of the prosthesis cement (for patients receiving topical TXA, we waited five minutes to allow the medication time for absorption). The treatment allocation was concealed from the patient, but not from the team performing the surgery and analyzing the data. Tissues were then closed and a suction drain (3.2 mm) was inserted.

Patients were allowed to control pain using a medication pump for 48 hours after surgery (4 ml per hour, 6 ml bolus, interval of 20 minutes between pumps and a maximum allowance of 60 ml in 24 hours) with self-administration of 165 ml of saline (0.9%), ropivacaine (20 ml, 1%), and fentanyl (15 ml). The infusion pumps were maintained with epidural infusion of propofol and opioids. The PCA pump for epidural infusion of analgesia included saline (0.9%, 95 ml) and morphine (50 mg, 1-2 mg/hour, 1 mg bolus every 15 minutes) with a maximum allowance of 6 mg/hour.

All patients received the same postoperative care protocol: physical therapy with continuous passive motion equipment, with a gradual increase in flexion, beginning with 60 degrees of knee flexion (1 hour, 3 times per day); prevention of venous thrombosis with elastic stockings, and sodium enoxaparin (Clexane®, Sanofi), 40 mg, administered subcutaneously once a day for 10 days.

All patients underwent duplex ultrasound examinations of the lower limbs on the 15th day after surgery, regardless of the presence of symptoms.

### Outcome and statistical analysis

This is a secondary analysis of data from a wider study that was conducted to verify the effects of TXA on blood loss and need for blood transfusion in knee surgery (the main study is registered on the clinicaltrials.gov database, under protocol record NCT02323373, and on the Plataforma Brasil, under the number 27270814.0.0000.0085). In this secondary analysis, we evaluated the prevalence of deep venous thrombosis (DVT) as detected or ruled out by duplex ultrasound scanning on the 15th day after surgery, regardless of symptoms.

We used SPSS software (version 13.0) for statistical analysis. Significance was set at 5%. We used the *t* test for paired samples, the chi-squared test, and the nonparametric Kruskal-Wallis test and the Mann-Whitney test (when outliers were found). Analysis of variance (ANOVA) with Bonferroni multiple comparisons was also used.

## RESULTS

**Figure 1:**
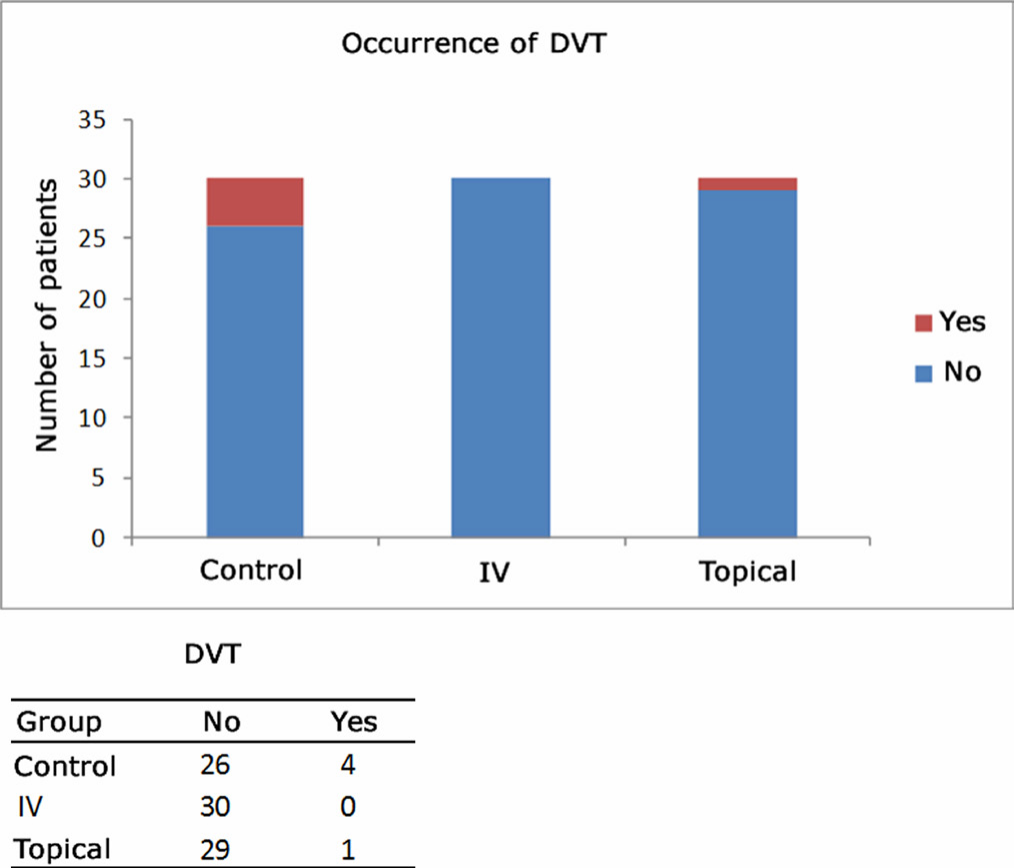
Post-operative evaluation of DVT occurrence/absence.

A total of 102 patients underwent TKA during the study period and were enrolled for the main study and for the secondary analysis. Twelve patients were excluded for reasons shown in Figure 1. Eighty patients were operated on with regional anesthesia (spinal and epidural) and 10 with general anesthesia. Preoperative hemoglobin levels were 13.5 g/dl in the control group, 13.9 g/dl in the intravenous group and 13.8 g/dl in the topical group, with no significant difference between groups.

There were no operative complications, no harmful effects or side effects, and no deaths or dropouts. The duration of surgery varied from 61 to 90 minutes, with an average time of 77 minutes. Tourniquets were used with fixed pressure of 300 mmHg and the duration of tourniquet use varied from 50 to 75 minutes, with an average of 63 minutes.

Mean patient age was 65.7 years (range: 48-88). Most patients were female (24 in the intravenous group, 21 in the topical group and 25 in the control group). The three groups were homogeneous in terms of age, sex and laterality (p > 0.05).

One case of DVT (3.3%) was observed in the topical group (in the gastrocnemius vein), no cases were detected in the intravenous group, and there were four cases (13.3%) in the control group (one in the popliteal vein and three in the gastrocnemius vein). These events were all diagnosed by duplex ultrasound scanning and the patients were all asymptomatic. These five patients were treated clinically by a vascular surgery specialist. The prevalence rates of DVT were similar between groups (p = 0.112 for control vs. intravenous; p = 0.353 for control vs. topical and p = 1.000 for intravenous vs. topical, according to two-sided exact tests).

## DISCUSSION

Tranexamic acid (TXA) has been used in heart and liver surgery for several years and more recently in hip and knee arthroplasties. Activation of fibrinolysis begins with the surgical trauma and TXA administered around 15 min before the tourniquet is applied delays fibrinolysis and reduces bleeding.^31^ However, the increased use of TXA in hip and knee arthroplasties raises concerns related to the safety of this medication in terms of blood clotting and the incidence of deep vein thrombosis (DVT).

Symptoms of DVT may include pain, calf edema or reduced blood perfusion. In our study, we determined occurrence/absence of DVT events in all patients by duplex ultrasound examinations on the 15th postoperative day, regardless of signs or symptoms such as lower venous return deficiency or calf muscle tenderness. We found 5 cases of DVT in 90 patients, just one of which was in the topical group. Therefore, no association was detected between use of the medication and incidence of DVT, whether via topical or intravenous administration. We took care to examine all patients by duplex ultrasound, even asymptomatic patients. Selvaratnam et al.^32^ assessed DVT using duplex ultrasound scanning after knee and hip arthroplasties, but only examined patients with symptoms. Shen et al.^33^ used duplex ultrasound scanning to examine all patients, but used a different dosage of the medication (15 mg/kg) given at different times to in our study (they used the drug before tourniquet release), with a smaller sample (45 patients) and they did not evaluate topical administration of TXA. Benoni et al. conducted a placebo-controlled study in which TXA was administered intravenously at the end of surgery (knee arthroplasty) and after three hours. The dosage used was 10 mg/kg in 50 ml of saline and plasminogen was measured in peripheral blood just before the operation, at the end of surgery, and three hours later. The study showed that administration of TXA reduced fibrinolysis in the wounds, but not in peripheral venous blood, which would explain the absence of DVT.^7^

As an antifibrinolytic agent, TXA could theoretically increase DVT prevalence. We therefore took care to evaluate all patients using duplex ultrasound scanning. Nevertheless, it so far seems that TXA is safe. The main advantages of topical administration of TXA are the possibility of using lower dosages^28^ and the absence of systemic absorption of the drug, which includes the possible risk of a hypercoagulable state,^34^ while delaying initiation of fibrinolysis.^9^^,^ 27 Tranexamic acid can also be administered by intra-articular injection, through the drain, immediately after closure of the surgical wound, with effects on total blood loss and knee joint swelling,^29^ but this technique was not evaluated here. Our study evaluated the incidence of DVT with duplex ultrasound scanning in all cases and examinations were performed after the point of peak DVT incidence, which is on the sixth day according to a study by Schiff et al.^25^ and when patients were no longer subject to the effects of the anticoagulant therapy.

Limitations of the present study include the small number of patients and the fact that double blinding was not possible because intravenous and topical administrations of a drug are obviously different from each other and so it would not have been possible to conceal experimental group allocation from the medical staff. Another limitation was that it was not possible to use a single examiner for duplex ultrasound scanning of all patients.

## CONCLUSIONS

The preliminary results of this study show that both topical and intravenous administration of tranexamic acid, used to reduce bleeding in TKA, are safe in regard to the prevalence of DVT, since the number of DVT cases was similar when patients receiving TXA or placebo were compared. Further studies, with larger samples, are needed to confirm this finding.
